# Preparation and Optical Properties of GeBi Films by Using Molecular Beam Epitaxy Method

**DOI:** 10.1186/s11671-017-2409-x

**Published:** 2017-12-20

**Authors:** Dainan Zhang, Yulong Liao, Lichuan Jin, Qi-Ye Wen, Zhiyong Zhong, Tianlong Wen, John Q. Xiao

**Affiliations:** 1 Center for Applied Chemistry, University of Electronic Science and Technology of China, Chengdu, 610054 China; 2 Department of Electrical and Computer Engineering, University of Delaware, Newark, DE 19716 USA; 3 Department of Physics and Astronomy, University of Delaware, Newark, DE 19716 USA

**Keywords:** GeBi films, MBE growth, Infrared properties, Terahertz properties

## Abstract

Ge-based alloys have drawn great interest as promising materials for their superior visible to infrared photoelectric performances. In this study, we report the preparation and optical properties of germanium-bismuth (Ge_1-x_Bi_x_) thin films by using molecular beam epitaxy (MBE). GeBi thin films belong to the n-type conductivity semiconductors, which have been rarely reported. With the increasing Bi-doping content from 2 to 22.2%, a series of Ge_1-x_Bi_x_ thin film samples were obtained and characterized by X-ray diffraction, scanning electron microscopy, and atomic force microscopy. With the increase of Bi content, the mismatch of lattice constants increases, and the GeBi film shifts from direct energy band-gaps to indirect band-gaps. The moderate increase of Bi content reduces optical reflectance and promotes the transmittance of extinction coefficient in infrared wavelengths. The absorption and transmittance of GeBi films in THz band increase with the increase of Bi contents.

## Background

In the field of optical communication, the optical wavelength in dense wavelength division multiplexing technology has extended from C-band (1.53–1.56 μm) to L-band (1.56–1.62 μm) at present. So the wavelength of optoelectronic detectors should include C-band and L-band. Because of emerging applications in the mid-infrared, however, the response cut-off wavelength of detectors should be longer than 2 μm. It is important to prepare semiconductor photoelectric detectors in near-infrared and far-infrared wave band with the wavelength in 2–10-μm range [1–4].

So far, Ge-based alloys were proved to be promising materials for infrared optoelectronic detectors. In 1984, AT& T.Bell Laboratories prepared GeSi film n-i-p devices by the molecular beam epitaxy (MBE) method, and the working wavelength was 1.45 μm [5, 6]. In 2010, the University of Stuttgart prepared GeSn films with 0.5–3% Sn content using low growth temperatures and pin detectors with 1.2–1.6-μm operating wavelength [7–10]. In 2011, academician Wang Qiming of the Chinese Academy of Sciences prepared the GeSn alloy with 1.0–3.5% Sn content and then successfully prepared pin detectors with working wavelength in the 1.3–1.6-μm range [11–13]. In 2014, M. Oehme developed GeSn/Ge multiple quantum well photodetectors with vertical structures, and the pin cut-off frequency was above 1.6 μm [14]. In 2015, S. Wirths successfully prepared GeSn films with direct band-gaps and prepared GeSn film detectors with 1.5-μm wavelength [15]. K. Toko prepared optoelectronic devices with 1.2–1.6-μm wavelength on flexible substrates by RF magnetron sputtering technology [16]. However, the cut-off wavelength of GeSi and GeSn semiconductor infrared detectors is still shorter than 2.0 μm, and the application wavelength cannot include the whole C-band and L-band. Finding new materials which have a longer cut-off wavelength will be useful to solve this problem.

Here, we report the preparation and optical properties of an n-type GeBi semiconductor thin film with longer cut-off wavelength by using the MBE method. The cut-off frequency was 2.3 μm, and the wavelength for applications was in the range from 1.44 to 1.93 μm, which includes both C-band and L-band. In this study, the effects of Bi alloying on the infrared and terahertz (THz) properties of Ge_1 − x_Bi_x_ films are investigated in details.

## Experimental Procedures

GeBi films were grown by using the MBE method with the vacuum pressure ranging from 4 × 10^−9^ to 5 × 10^−10^ Torrs. Ge atoms and Bi atoms were ejected from a Ge source (1200 °C) and a Bi source (400–550 °C), respectively, which arrived at the (100) substrate surface of a p-type Si single crystal wafer, and formed the films finally. The substrate temperature was 150 °C, and the growth rate ranged from 1.66 to 2.50 nm/min. The detailed growth parameters of the GeBi films are summarized in Table 1.

**Table 1:** Growth parameters of GeBi films

Sample no.	Injection temperature	Substrate temperature (°C)	Ge_1-x_Bi_x_
1	1200 °C/400 °C	150	Ge_0.980_Bi_0.020_
2	1200 °C/425 °C	150	Ge_0.898_Bi_0.102_
3	1200 °C/450 °C	150	Ge_0.817_Bi_0.183_
4	1200 °C/475 °C	150	G_e0.797_Bi_0.203_
5	1200 °C/500 °C	150	Ge_0.778_Bi_0.222_

The phase formation of GeBi films was characterized by grazing incident X-ray diffraction (XRD). The morphology of the GeBi films was analyzed by a scanning electron microscope (SEM; JMS6490LV, JEOL, Tokyo, Japan). The roughness of the samples was tested by atomic-force microscopy (AFM, 300 HV, SEIKO, Japan). Raman spectroscopy was tested by a Raman spectrometer (LabRAM HR, Edison, NJ, USA). The near-infrared and far-infrared properties of the GeBi films were measured by an optical spectrometer (Lambda 75UV/VIS/NIR) and a far-infrared spectrometer. The THz wave transmission properties were measured by THz time domain spectroscopy.

## Results and Discussion

**Fig. 1:**
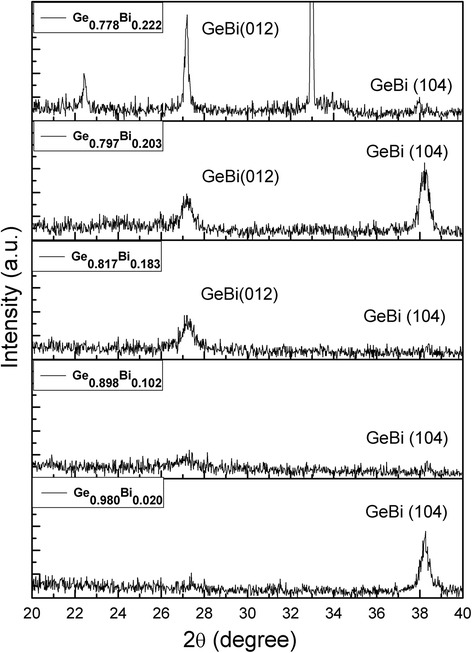
XRD patterns of Ge_1 − x_Bi_x_ film GeBi films with different Bi contents ranging from *x* = 0.020 to *x* = 0.222

Figure 1 shows XRD patterns of the as-prepared Ge_1 − x_Bi_x_ films. It can be seen that characteristic diffraction peaks which can be attributed to GeBi alloys can be found in all the MBE growth samples. Figure 1 shows the XRD results of the Ge_1 − x_Bi_x_ films grown by MBE without thermal treatment. All the samples show the diffraction peaks of GeBi film, while the crystalline property of the samples varies when the Bi content (*x*) changes from 0.020 to 0.222. When the Bi content is low (*x* = 0.020), the Ge_0.980_Bi_0.02_ film was found to be oriented along the (014) direction, see Fig. 1. With the Bi content increased to *x* = 0.102, beside the (104) peak locating around 2*θ* = 38.2^o^, the (012) peak of GeBi film locating around 2*θ* = 27.2^o^ starts to appear. With increasing Bi content (*x*) from 0.183 to 0.222, the intensity of (012) peak was dramatically increased while the (104) peak almost disappeared. This indicates the Ge_1 − x_Bi_x_ films with higher Bi content were preferably oriented along the (012) direction instead of the (104) direction. The different contents of Bi had an influence on the microstructures of the films. For the GeBi films with different Bi contents, changing the growth parameters could influence the preferred orientation of growth. We speculate that because of the low melting point of Bi atoms, Bi atoms formed groups with Ge atoms, entered into crystal lattices, and formed Ge-Bi cells. XRD results indicate that GeBi films were successfully prepared by MBE method and their crystalline properties could be manipulated by changing the Bi content in the Ge_1 − x_Bi_x_ films.

**Fig. 2:**
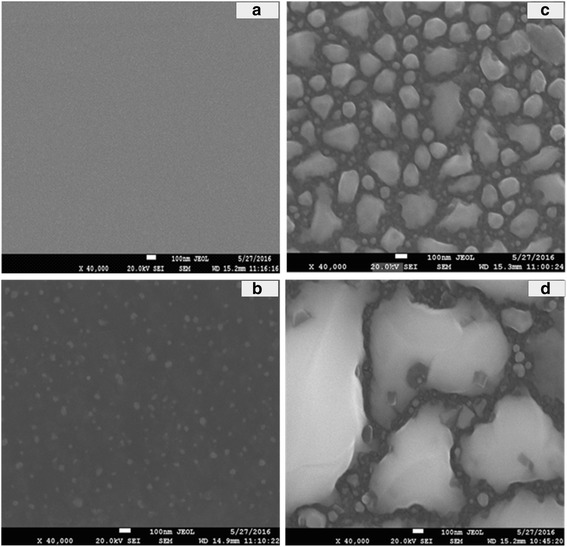
Typical SEM patterns of GeBi films with different Bi contents: **a** 2.0%; **b** 10.2%; **c** 18.3%; and **d** 20.3%

Typical SEM images of the Ge_1 − x_Bi_x_ film samples are presented in Fig. 2. When the Bi content was 2.0% (*x* = 0.02), the GeBi film grew well and its surface was found very smooth, see Fig. 2a. When the Bi content increased to 10.2%, there were some small dots in homogeneous media, which was the expression of the initial forming process of new phases, see Fig. 2b. Because of the lowest energy principle, superficial Bi atoms segregated and aggregated into groups (size 33–42 nm). When the Bi content reached over 18.3%, there were three phases in the film at least, such as GeBi, amorphous Bi, and Ge, see Fig. 2c, d. The grain size of GeBi films was very big, up to about 1000 nm. The segregated Bi and Ge particles, with a small grain size in the range from 30.7 to 118.0 nm, were found between the crystal boundaries of GeBi grains. We found that, when the Bi content over the solid solubility in the GeBi alloy, excessive Bi atoms would be deposited and formed Bi phases at the large grain boundary under low temperatures. Some Ge atoms, which could not react with Bi atoms due to the restriction of the low temperature, also formed the Ge phase at the large grain boundary. Nevertheless, the increase of the Bi content could have promoted the preferred growth of GeBi grains, and the grain sizes changed from 42 to 100 nm, see Fig. 2b, d.

**Fig. 3:**
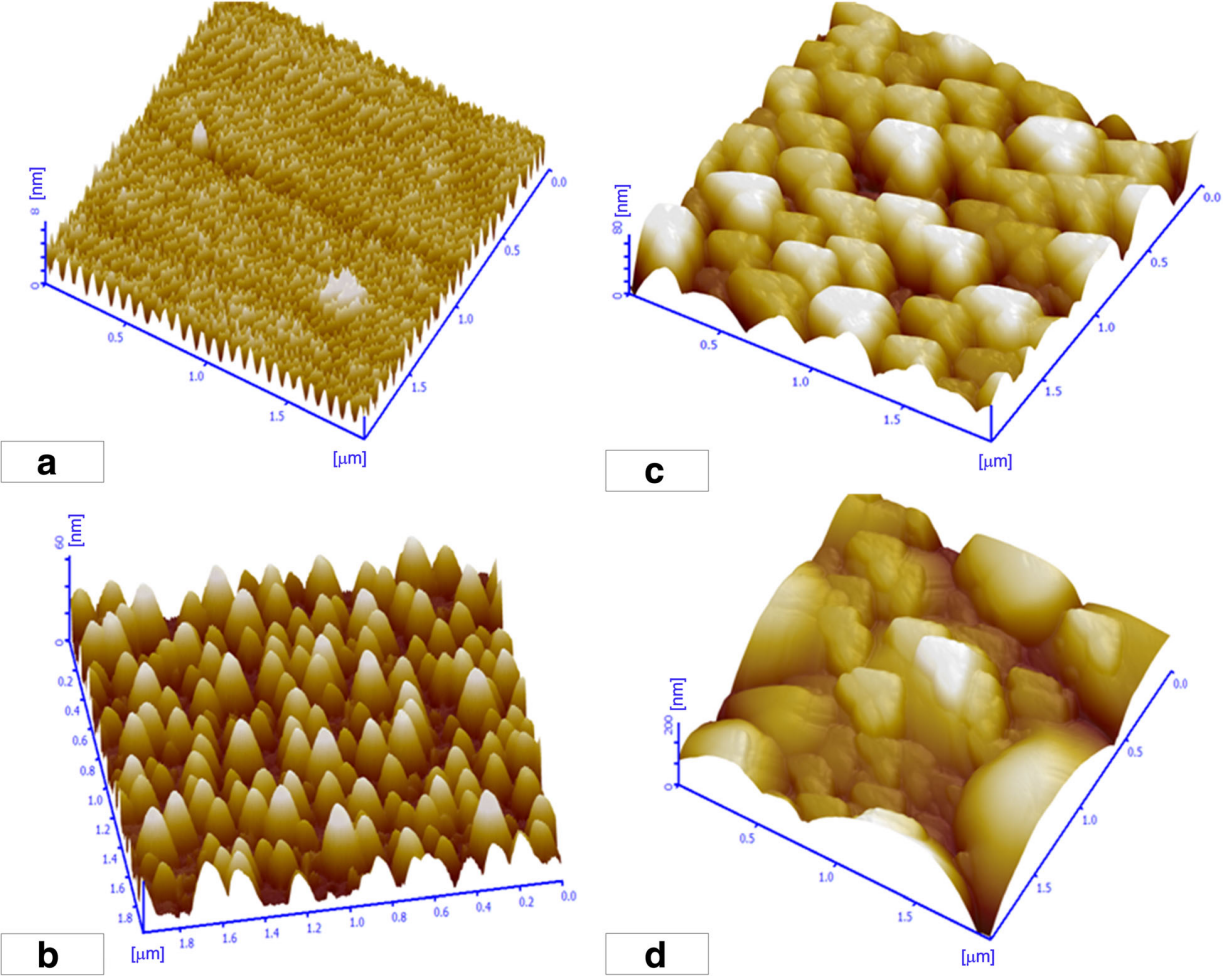
AFM test results of GeBi films with different Bi contents: **a** 2.0%; **b** 10.2%; **c** 18.3%; and **d** 20.3%

Figure 3 shows typical AFM images of the Ge_1 − x_Bi_x_ films with various Bi contents, and the Ra value and RMS values are summarized in Table 2. With the increasing content of Bi, the Ra value and RMS values increased drastically, indicating the surface roughness of the Ge_1 − x_Bi_x_ films is increased. Meanwhile, there were some irregular peaks in Fig. 3b–d due to the heterogeneous grain size and small grains in the grain boundaries. When the content of Bi was excessive, the number of Bi atoms replaced with Ge atoms was limited due to the restriction of solid solubility of Bi in the GeBi alloy. Supernumerary Bi atoms deposited on the film made the films rough and had a great influence on the microstructure of GeBi films, which is consistent with the SEM results.

**Table 2:** AFM test results of GeBi films with different Bi contents

Sample No.	Ra (nm)	*P*-*V* (nm)	RMS (nm)	Large cell (nm)		Small cell (nm)	
				Width	Height	Width	Height
1	1.013	9.311	1.196	53.39	3.9	47.67	1.4
2	9.809	62.84	11.61	165.07	31.3 m	66.79	14.0
3	12.44	95.45	15.76	636.35	42.2	307.02	35.0
4	37.06	291.30	45.65	947.27	161.8	439.10	48.5

**Fig. 4:**
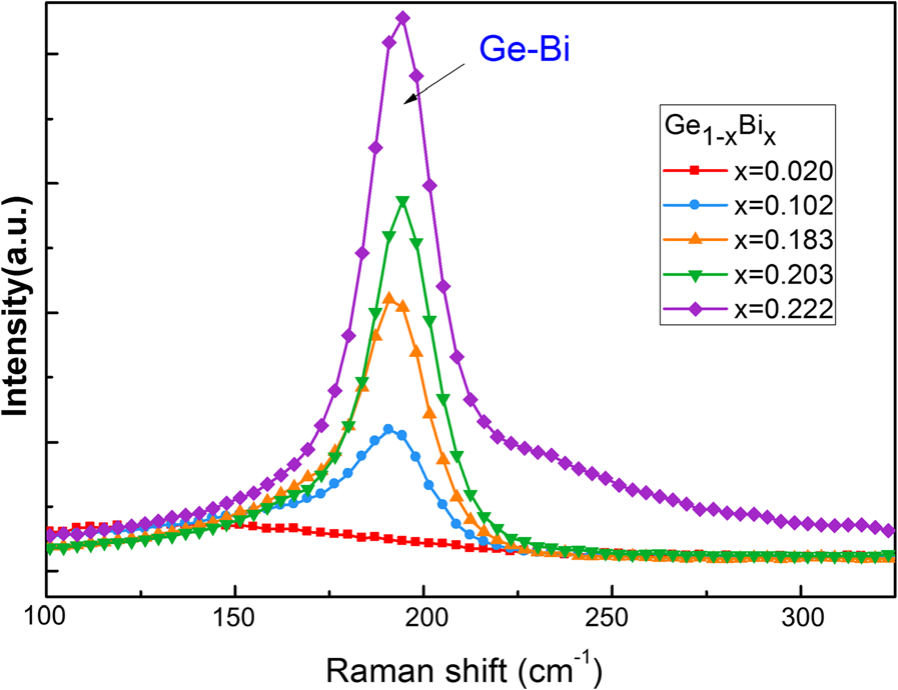
Raman spectra of GeBi films with different Bi contents

Figure 4 shows room temperature Raman spectra of the as-grown Ge_1 − x_Bi_x_ films with different Bi contents prepared by MBE. A serial of peaks located at around 190 cm^−1^ could be attributed to the Ge-Bi vibration mode. With increasing Bi content, the Ge-Bi peak became stronger and shifted toward to higher wavenumber (cm^−1^). The shift to higher wavenumbers indicated that, with the increase of Bi content, the mismatched rate of lattice constants of films and the lattice strain in GeBi films increased. It can be concluded that Bi doping is an effective way to tune the lattice strain of Ge_1 − x_Bi_x_ alloy thin films.

**Fig. 5:**
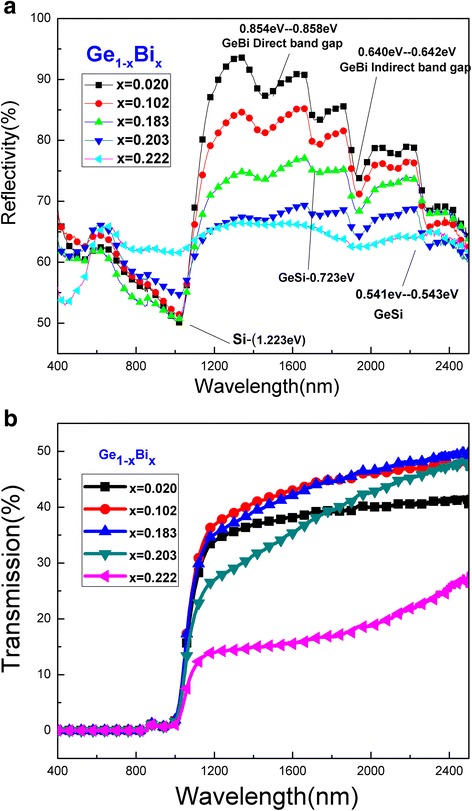
Reflectance spectra (**a**) and transmission spectra (**b**) of GeBi films with different Bi contents in near-infrared wave band

Figure 5 shows near-infrared properties of the GeBi films with different Bi contents. The absorption behaviors of the films were obtained from their reflectivity and transmission properties. As shown in Fig. 5a, with increasing Bi content, the reflectance of GeBi films reduced in the range of 1014–2500 nm, which indicated that absorption of films increased. The valley in the range of 1932–1938 nm could be attributed to indirect band-gaps absorption of the GeBi films. And the depth of energy absorbing valley reduced with increasing the Bi content. When the Bi content was more than 20%, the valley disappeared in the range of 1932–1938 nm. Direct band-gaps of GeBi films were in the range of 1446–1452 nm; the depth of energy absorbing valley also reduced with increasing the Bi content. When the Bi content was above 20.3%, the valley disappeared in the range of 1446–1452 nm. In conclusion, the increase of Bi content reduces the reflectance of GeBi films, increases the extinction coefficient, and makes the reflected amplitudes decreases finally. As shown in Fig. 5b, there was an inflection point at around 1020 nm (1.22 eV), which was attributed to the forbidden band-gap of Si at 1.12 eV. When the wavelength was less than the value of the inflection point, the transmittance of GeBi films and the Si substrate was small. In the 1020–2500-nm range, the transmittance increased with the increased wavelength. However, when the Bi content increased from 18.3 to 22.2%, the transmittance was reduced. In the 800–1600-nm range, tremendous changes of the index of refraction, the extinction coefficient, and the excessive Bi content had an effect on the absorption of films [17, 18].

**Fig. 6:**
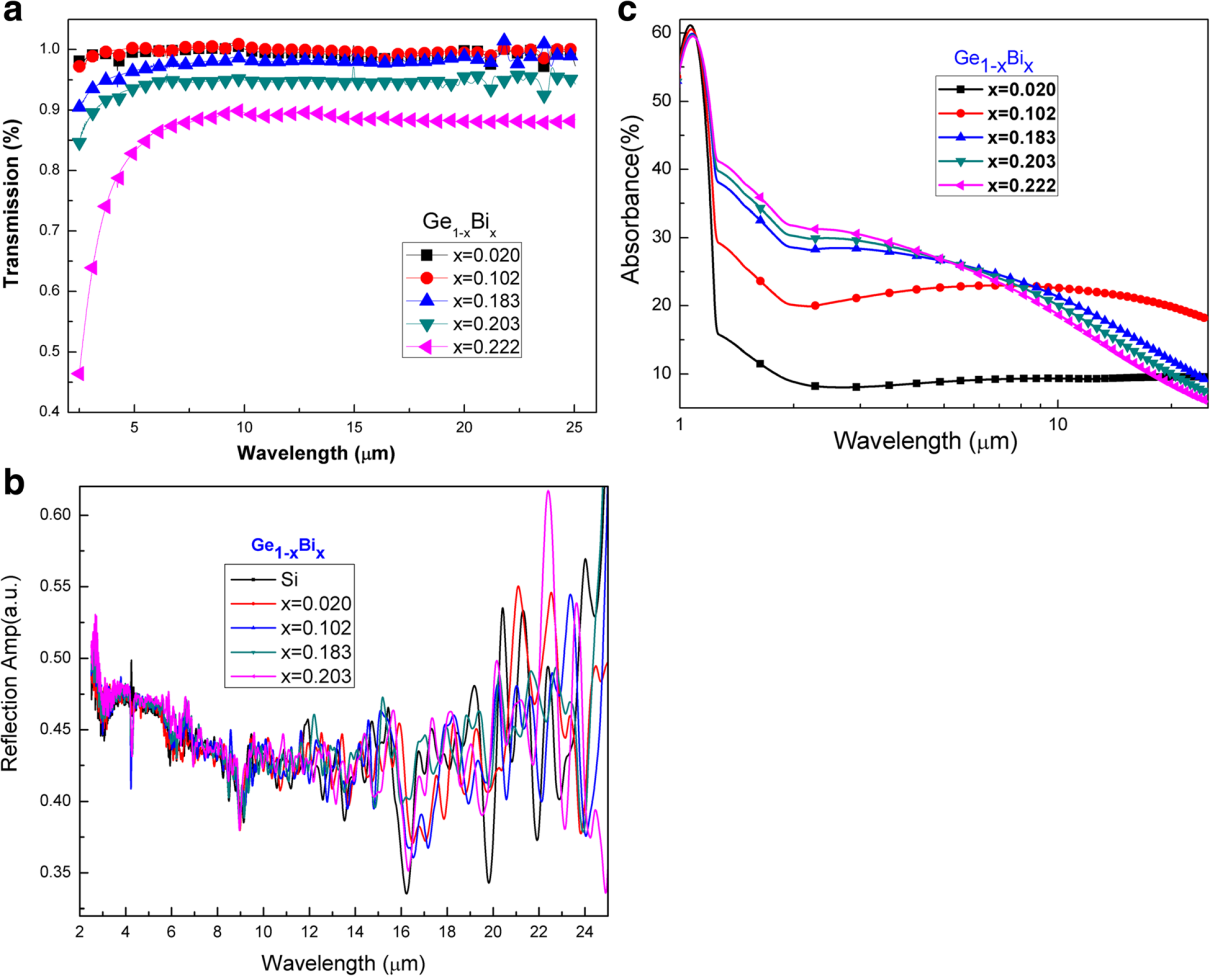
Transmission spectra (**a**), reflectance spectra (**b**), and absorption spectra (**c**) of GeBi films with different Bi contents in far-infrared wave band

**Fig. 7:**
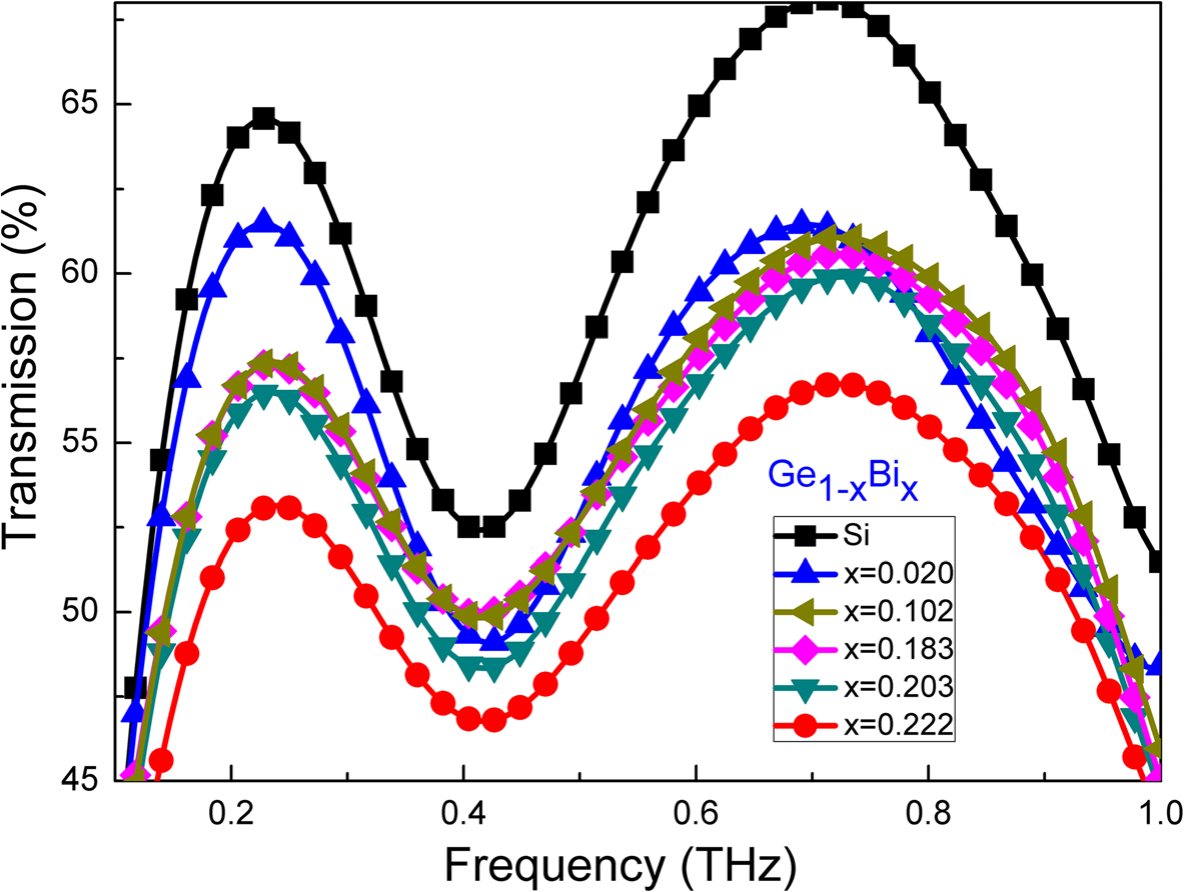
THz transmission spectra of GeBi films with different Bi contents

Figure 6 shows properties of the GeBi films with various Bi contents in far-infrared wave band. There was a high and stable absorption window for GeBi films in the 4–15-μm wave band, see Fig. 6a, b. Because the principles of reflectance and transmittance were different, we could not directly get the absorption of GeBi films from Fig. 6a, b. We analyzed the refraction and experimental results of the extinction coefficient of Ge films in the 1–25-μm wave band [17], considered the effect of the Bi content on the Ge films, and got the absorption spectra of GeBi films in far-infrared band finally, see Fig. 6c. With the increasing Bi content from 2 to 10.2%, the absorption increased from 9.3 to 22.6% in the range from 1 to 25 μm. The absorption had the same tendency with further increasing the Bi content. However, when the Bi content increased, the absorption of the Ge_1 − x_Bi_x_ thin films increased in the range from 1.0 to 7.5 μm and then decreased in 7.5–25-μm range. The Bi content above 10% resulted in the Bi atoms deposited in GeBi films, the surface roughness increased, and then, the absorption was reduced. Figure 7 shows the THz transmittance as a function of frequency for the GeBi films with different Bi contents. When the Bi content increased from 2 to 10.2%, the transmittance decreased 10%. The transmittance increased slightly with the Bi content increasing from 18.3 to 22.2%. The transmission measurements indicate that the THz properties of the Ge_1 − x_Bi_x_ thin films could be tuned by varying the Bi content, which is very important for the application such as THz wave modulators [19].

## Conclusion

In summary, Ge_1 − x_Bi_x_ films with Bi fraction *x* = 0 to 0.222 were successfully grown on p-Si (100) substrates by using low-temperature MBE. XRD and SEM results indicated that their crystalline and morphological properties could be manipulated by changing the Bi content in the Ge_1 − x_Bi_x_ films. The influences of the Bi content on the optical properties including the infrared and THz performances of the Ge_1 − x_Bi_x_ films were systematically investigated. The moderate increase of the Bi content reduced the reflectance and varied the transmittance in infrared wavelengths. The transmittance of GeBi films in THz band decreased with the moderate increase of Bi content. Thus, it can be concluded that the MBE Ge_1 − x_Bi_x_ films are promising materials for both infrared and THz applications.

## Abbreviations

AFM: Atomic-force microscopy
MBE: Molecular beam epitaxy
SEM: Scanning electron microscope
THz: Terahertz
XRD: X-ray diffraction
